# Action Taken by His Colleagues, &c.

**Published:** 1889-04

**Authors:** 


### THE FOLLOWING ACTION IN RELATION TO THE EDITOR'S LOSS WAS TAKEN BY HIS COLLEAGUES OF THE SOUTHERN MEDICAL COLLEGE.

DR. R. C. WORD,

CITY.

DEAR DOCTOR:—At a meeting of the Faculty of the Southern Medical College, the following was adopted as expressing the sentiments of the Faculty, and the Secretary was instructed to spread the same on the minutes and send a copy of the same to you.

"The colleagues of Dr. R. C. Word, in the Southern Medical College, realizing the great loss sustained in the destruction of his residence by fire, and being impressed with the inconvenience and distress thus brought upon his family, feel prompted to express a profound sympathy in their suffering from this calamity.

We would not expect that our friend and colleague can experience any greater deprivation in the loss of worldly goods than to be thus suddenly forced away from the comforts of home by the ruthless flames. But we regret to learn that along with these inconveniences he has sustained another irreparable loss in the destruction of the fruits of his literary and scientific labors.

We are deeply sensible of the magnitude of the loss in being thus deprived of the accumulated treasures of the mind. And we become partners in his sacrifice by the fact that it must involve immense additional labor in co-operating for the advancement of our interests in the Southern Medical College. In view of these facts and considerations, we hereby tender to Prof. R. C. Word and his entire family our heartfelt sympathy in their distress, with an earnest desire to promote their comfort and well being by all the means available to us."

Signed, W. D. BIZZELL, SEC.

PROF. W. D. BIZZELL, SECT'Y—

DEAR SIR:

I am pleased to acknowledge the receipt of your favor, communicating to me the action of the Faculty in relation to my recent misfortune by fire. Please say to my colleagues that I most warmly appreciate the words of kindness and sympathy so well and courteously expressed in my behalf.

It is especially gratifying, as evincing a feeling of brotherhood between the members of our Faculty, which is not only touching and appropriate, but eminently conducive to the furtherance of the great and useful enterprise in which we are engaged.

Gratefully and Respectfully,

R. C. WORD, M. D.

